# Pyrethroid-Resistance and Presence of Two Knockdown Resistance (*kdr*) Mutations, F1534C and a Novel Mutation T1520I, in Indian *Aedes aegypti*

**DOI:** 10.1371/journal.pntd.0003332

**Published:** 2015-01-08

**Authors:** Raja Babu S. Kushwah, Cherry L. Dykes, Neera Kapoor, Tridibes Adak, Om P. Singh

**Affiliations:** 1 National Institute of Malaria Research, Sector 8, Dwarka, Delhi, India; 2 School of Life Sciences, Indira Gandhi National Open University, Maidangarhi, New Delhi, India; Mahidol University, Thailand

## Abstract

**Background:**

Control of *Aedes aegypti*, the mosquito vector of dengue, chikungunya and yellow fever, is a challenging task. Pyrethroid insecticides have emerged as a preferred choice for vector control but are threatened by the emergence of resistance. The present study reports a focus of pyrethroid resistance and presence of two *kdr* mutations—F1534C and a novel mutation T1520I, in *Ae. aegypti* from Delhi, India.

**Methodology/Principal Findings:**

Insecticide susceptibility status of adult-female *Ae. aegypti* against DDT (4%), deltamethrin (0.05%) and permethrin (0.75%) was determined using WHO's standard insecticide susceptibility kit, which revealed resistance to DDT, deltamethrin and permethrin with corrected mortalities of 35%, 72% and 76% respectively. Mosquitoes were screened for the presence of *kdr* mutations including those reported earlier (I1011V/M, V1016G/I, F1534C, D1794Y and S989P), which revealed the presence of F1534C and a novel mutation T1520I. Highly specific PCR-RFLP assays were developed for genotyping of these two mutations. Genotyping using allele specific PCR and new PCR-RFLP assays revealed a high frequency of F1534C (0.41–0.79) and low frequency of novel mutation T1520I (0.13). The latter was observed to be tightly linked with F1534C and possibly serve as a compensatory mutation. A positive association of F1534C mutation with DDT and deltamethrin resistance in *Ae. aegypti* was established. However, F1534C-*kdr* did not show significant protection against permethrin.

**Conclusions/Significance:**

The *Aedes aegypti* population of Delhi is resistant to DDT, deltamethrin and permethrin. Two *kdr* mutations, F1534C and a novel mutation T1520I, were identified in this population. This is the first report of *kdr* mutations being present in the Indian *Ae. aegypti* population. Highly specific PCR-RFLP assays were developed for discrimination of alleles at both *kdr* loci. A positive association of F1534C mutation with DDT and deltamethrin resistance was confirmed.

## Author Summary

Dengue and chikungunya are the two important human arboviral infections in India transmitted mainly by *Aedes aegypti*. In absence of any specific drug or vaccine for these infections, vector control and personal protection are the only control options available. The success of insecticide-based vector control heavily relies upon the knowledge of the status of insecticide resistance in vector populations and the underlying mechanisms of insecticide resistance, especially in the presence of cross-resistance. Knockdown resistance (*kdr*) is one of the mechanisms of resistance that confers cross-resistance to DDT and pyrethroids. Currently, pyrethroids are the only insecticide class recommended for use in long-lasting insecticide nets (LLIN) and have proven superior to all other insecticides used in vector control programme, due to low mammalian toxicity, low residual activity in nature and rapid knockdown action. The present study was undertaken to determine the susceptibility status of *Ae. aegypti* against DDT and pyrethroids, and identification of *kdr* mutations. Though the presence of *kdr* mutations in *Ae. aegypti* has been reported in many countries, such a report is not available from India. This study for the first time reports the presence of two *kdr* mutations, F1534C and a novel mutation T1520I, in an Indian *Ae. aegypti* population.

## Introduction

*Aedes aegypti* is globally distributed throughout the tropics and subtropics and highly adapted to humans and urban environments. It acts as a primary vector for various arboviral infections including yellow fever virus, dengue virus (DENV) and chikungunya virus (CHIKV) [1]–[5]. Dengue has recently become a major health problem around the world with more than 120 countries endemic for dengue [6] and has been ranked as the most important mosquito borne viral disease [7]. Recent estimates by the World Health Organization (WHO) suggests that 50–100 million dengue infections occur worldwide every year and over 40% of the world's population is now at risk of the disease [8]. A study based on a cartographic approach estimated 90 million apparent dengue infections globally in year 2010 with India accounting for 34% (32 million) infections [9]. Chikungunya is another important arboviral infection spread by *Ae. aegypti*, prevalent in Africa, Southeast Asia and India [10]. In India it re-emerged in 2006 after a gap of 32 years [10].

Since there is no specific vaccine or drug available for the treatment of dengue and chikungunya, vector control and personal protection are the only options to reduce the spread of these arboviral infections. Vector control strategies employed for *Aedes* control in India are mainly anti-larval measures, source reduction and use of adulticides (pyrethrum space spray and malathion-fogging) during a disease outbreak. Pyrethroids are widely used for personal protection in the form of repellents and insecticide treated materials [11], which provides effective protection against day biting *Aedes*. It has also been shown that window curtains and domestic water container covers treated with insecticide may reduce densities of dengue vectors to low levels and potentially affect dengue transmission [12]. In addition pyrethroids have been recommended by WHO for space spraying for *Aedes* control [13] due to rapid knockdown effect and less mammalian toxicity. However, the use of pyrethroids is being challenged by the rapid emergence of resistance, which needs to be monitored periodically to manage effective programmes to avoid or delay resistance in vector species. Key to this is, understanding of the mechanisms of resistance so that informed decisions can be made to select appropriate insecticides for effective control of target vector species.

One of the mechanisms of resistance in insects against DDT and pyrethroids is knockdown resistance (*kdr*) which is conferred by mutation(s) in the target site, the voltage gated sodium channel (VGSC). Several *kdr* mutations have been reported in many insects of agricultural and medical importance including *Ae. aegypti*. In *Ae. Aegypti*, eleven non-synonymous mutations at nine different loci have been reported [14]–[17], amongst which mutations at three loci, i.e., Iso1011 (I→M/V) and Val1016 (V→G/I) in domain II and F1534 (F→C) in domain III are most commonly reported as contributing to pyrethroid resistance [14]–[22]. The most common *kdr*-mutations L1014F/S reported in many insects of agricultural and medical importance is not yet found in *Ae. aegypti* possibly due to codon constraint [23]. Although widespread in Southeast Asia and Latin America, the presence of *kdr* mutations has yet to be established in India. Here we report the presence of two *kdr* mutations, F1534C and a novel mutation T1520I, in an Indian *Ae. aegypti* population.

## Materials and Methods

### Mosquito collection

*Aedes* immature (larvae and pupae) were collected from the water holding containers in domestic and peri-domestic areas in Delhi and were reared to adults. The collection sites and dates of collections are shown in supplemental items S1 Table and S1 Fig. Ground mixtures of dog biscuits and fish food in a ratio of 3∶1 were provided as food for larvae. Emerged adults were identified morphologically and supplied with 10% glucose solution soaked in cotton pads.

### Insecticide susceptibility bioassay

Two-to four-days old adult *Ae. aegypti* female mosquitoes were subjected to insecticide susceptibility testing using the WHO's standard insecticide susceptibility test kit. Up to twenty-five mosquitoes in each replicate were exposed to 4% DDT, 0.05% deltamethrin and 0.75% permethrin impregnated paper (supplied by WHO collaborative centre, Vector Control Research, Universiti Sains, Malaysia) alongside appropriate controls for one hour and subsequently transferred to recovery tubes lined with untreated paper. During recovery, mosquitoes were provided access to cotton soaked in 10% glucose and mortalities were recorded after 24 hours. All the bioassays were carried out at 27±1°C and 70±10% relative humidity. Percent mortalities were calculated using Abbott's formula [24]. Dead and alive mosquitoes after recovery was transferred to individual microfuge tubes and stored at −20°C.

### DNA isolation and *kdr* genotyping

DNA was isolated from individual mosquitoes following Livak *et al.* (1984) [25]. Allele specific PCR assays were employed for genotyping of *kdr* mutations I1011V/M, V1016G/I and F1534C following Saavedra *et al.* (2007) [16] and Yanola *et al.* (2011) [26]. For genotyping of D1794Y, PCR-RFLP was carried out as described by Chang *et al.* (2012) [27]. In the absence of established PCR-based assays for mutation S989P, direct sequencing was carried out using primers IIP_F and IIS6_R [26]. Dead as well as surviving mosquitoes of some batches of insecticide bioassay tests were genotyped for F1534C mutation to study the association of this *kdr* mutation with insecticide resistance.

### DNA sequencing

DNA sequencing was performed to validate the PCR-based genotyping used for various *kdr* alleles and also to check for the presence of any novel mutation. Three regions of VGSC were amplified and sequenced: (i) partial domain II (P to S6) using primers IIP_F and IIS6_R [26], (ii) partial domain III (S4–S6) using primers Ge-IIIS6_F and IIIS6R [26], and (iii) partial domain IV (S5–S6) using primers 5380F1 and 5380R1 [27]. PCR products were amplified, purified using QIaquick PCR purification kit (Qiagen Inc) and subjected to cycle sequencing reaction using BigDye Terminator v3.0. The termination products were run in Applied Biosystems 3730×l DNA Analyzer. Some of the sequencing reactions were performed at Macrogen Inc (South Korea). Sequencing chromatograms were edited using FinchTV ver 1.5.0 (Geospiza, Inc.). The PCR product of one sample, which was suspected to have an indel in the intron, was cloned in pGEM-T vector system using vendor's protocol and five clones were sequenced. Sequences were aligned using ClustalW implemented in Mega5 [28].

### Development of PCR-RFLP for *kdr* genotyping

For development of PCR-RFLP assays for detection of *kdr* alleles at two loci (F1534 and T1520) in domain III-S6, DNA sequences spanning 200 bp upstream to F1534 and 200 bp downstream to T1520 were checked for 1534C- and 1520I-specific restriction sites using an online tool available at http://insilico.ehu.es/restriction/two_seq. Two unique restriction enzymes *Ssi*I and *BsaB*I were selected which were specific to1534C (TTC>TGC) and 1520I (ACC>ATC) sequences respectively. The intron region was excluded when designing PCR-RFLP due to the existence of indel in the intron upstream of T1520 as revealed by sequencing of cloned PCR product. Two primers flanking these two loci, i.e., AekdrF (5′-TGGGAAAGCAGCCGATTC-3′) and AekdrR (5′-CCTCCGTCATGAACATTTCC-3′) were designed with expected amplicon size of 171 bp. The expected sizes of cleaved product for the 1520I allele were 143 and 28 bp when digested with *BsaB*I, and 103 and 68 bp for 1534C when digested with *SSi*I. The diagnostic criterion for 1520I allele was taken as the presence of 143 bp band only (resolution of 28 bp cleaved product can not be resolved on agarose gel), whereas presence of 103 and 68 bp bands were considered as diagnostic criteria for 1534C allele. Uncut product of 171 bp was considered the wild allele.

**Figure 1:**
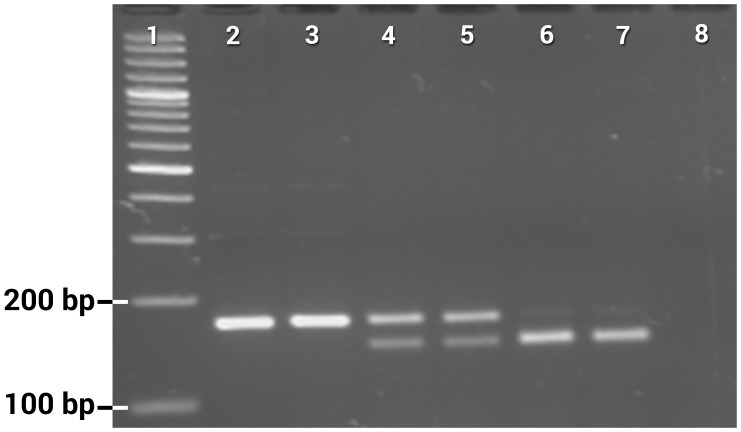
Gel photograph showing PCR-RFLP assay for genotyping of T1520 alleles. Lane 1: 100 bp DNA ladder, lanes 2–3: TT, lanes 4–5: TI heterozygotes, lanes 6–7: II, lane 8: negative control.

**Figure 2:**
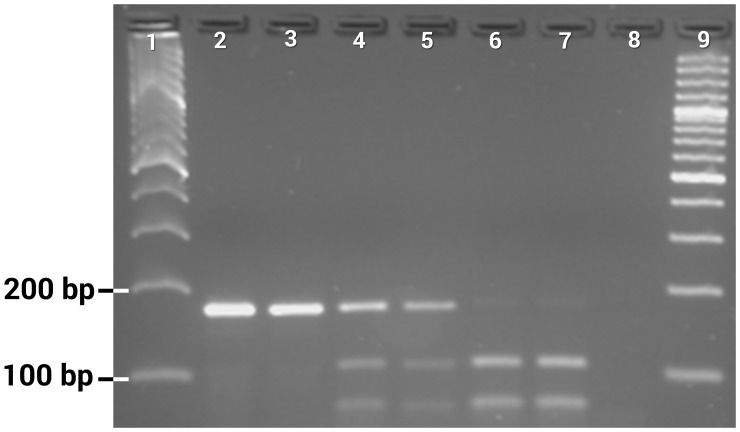
Gel photograph showing PCR-RFLP assay for genotyping of F1534 alleles. Lanes 1 and 9: 100 bp DNA ladder, lanes 2–3: FF, lanes 4–5: FC heterozygotes, lanes 6–7: CC, lane 8: negative control.

For PCR-RFLP, amplification was carried out in 15 µl of reaction mixture containing 1× buffer, 200 µM of each dNTP, 0.25 µM of primers AekdrF and AekdrR and 0.5 unit of *Taq* DNA polymerase. The PCR conditions were initial denaturation at 95°C for 3 min followed by 35 cycles each of denaturation at 95°C for 15 s, 50°C for 15 s and extension at 72°C for 30 s and a final extension at 72°C for 7 min. The PCR product was subjected to two separate restriction digestion reactions, one with *BsaB*I and another with *SSi*I. Each restriction digestion reaction mixture (20 µl) contained 5 µl of PCR product, 2 units of restriction enzyme and 1× buffer, which was incubated for four hours or overnight at 65°C for *BsaB*I and 37°C for *SSi*I. The cleaved product was run on 2.5% agarose gel containing ethidium bromide and visualized with a gel documentation system (Figs. 1 and 2).

Representative samples of PCR-RFLP genotyped samples were sequenced for partial domain III to validate PCR-RFLP result where primers AekdrF and AekdrR were used for amplification of PCR product and only AekdrR was used for sequencing reaction.

DNA sequences with read length of 200 bp or more have been deposited in GenBank (accession numbers: KM677247–KM677334).

### Statistical analysis

The association of the *kdr* mutations with resistance phenotype was tested using Fishers' Exact test and Odds Ratio estimation using dominant, recessive and additive models. Hardy-Weinberg equilibrium test was performed using Chi-square or Fisher's Exact test. Analysis of linkage disequilibrium and the Hill and Robertson coefficient *r* ^2^ were calculated for alleles using CubeX software (http://www.oege.org/software/cubex) [29].

## Results

### Insecticide susceptibility status

The result of insecticide susceptibility tests carried out on *Ae. aegypti* against DDT, deltamethrin and permethrin are shown in Table 1. The result shows high resistance against DDT (30.2–48.1% mortality) and moderate level of resistance to pyrethroids (deltamethrin: 64.4–74.3%; permethrin: 66.8–82.3% mortalities) in all sites.

**Table 1:** Result of insecticide susceptibility test against DDT, deltamethrin (DEL) and permethrin (PER), and genotyping result of F1534 alleles as determined by allele specific PCR.

Locality	Percent corrected mortality (replicates/n)			F1534 genotypes				Allelic frequencies		*p* * (HWE)
	DDT 4%	DEL 0.05%	PER 0.75%	FF	FC	CC	Total	F1534	1534C	
South Delhi I	30.17% (20/348)	71.86% (12/231)	82.31% (19/373)	118	195	214	527	0.409	0.591	0.000
South Delhi II	37.58% (9/165)	74.32% (8/148)	66.79% (14/265)	35	128	158	321	0.308	0.692	0.504
West Delhi	48.15% (6/108)	64.41% (3/59)	74.74% (5/95)	139	112	81	332	0.587	0.413	0.000
Pooled data	35.27% (35/621)	71.69% (23/438)	75.72% (38/733)	292	435	453	1180	0.432	0.568	0.000

HWE = Hardy-Weinberg equilibrium

*chi-square test.

### Genotyping of *kdr* alleles

Results of allele specific PCR genotyping for the F1534C mutation are shown in Table 1. The allelic frequency of the 1534C mutant is high in all the three sites ranging from 41—69%. Of the1180 samples genotyped, a total of 34 sample representing FF (n = 9), FC (n = 11) and CC (n = 14) were sequenced for partial domain III to validate allele specific PCR results. Two samples showed discrepancies where homozygous CC turned out to be FC after sequencing. Sequencing of samples also revealed the presence of a novel mutation C>T on the second codon of T1520 residue (ACC) leading to T→I amino acid substitution. Among 34 samples sequenced for partial domain III, eleven were with FC/TT, two with FC/TI, eleven with CC/TT, one with CC/II and the remaining nine were with FF/TT.

A total of 166 samples were genotyped for I1011M/V and V1016G/I. Though some samples were observed to be positive for mutations (genotypes IM = 7, MM = 1, IV = 2 for I1011 locus, and VI = 6 for V1016 locus) by allele specific PCR, sequencing of 29 samples representing all genotypes (all mutants and 13 wild genotypes) did not confirm the presence of any of them. Sequencing of partial domain II also did not identify S989P-*kdr* mutation in any sample. The genotyping for I1011 and V1016 was therefore discontinued assuming that allele specific PCR is not specific and I1011M/V or V1016G/I mutations are absent in the study population. For genotyping of D1794Y, a total of 66 mosquitoes were genotyped using PCR-RFLP and five samples through DNA sequencing, but all turned out to be the reference genotype.

### Association of F1534C mutation with insecticide resistance

**Figure 3:**
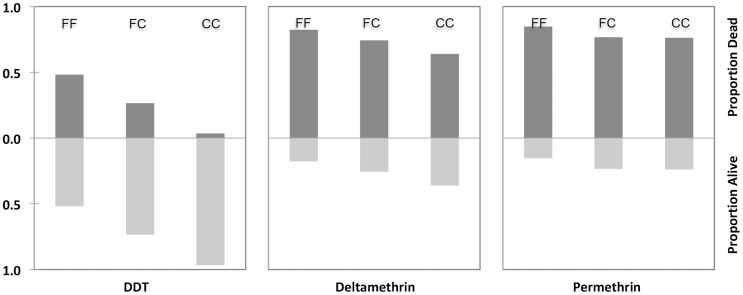
Proportion of dead and alive mosquitoes in each genotype for F1534 alleles exposed to DDT 4%, deltamethrin 0.05% and 0.75% permethrin for one hour.

The distribution of different F1534 is shown in Table 2. The proportions of dead and live mosquitoes after exposure to insecticides for each genotype are shown in Fig. 3. Odds Ratio (OR) estimates at 95% confidential intervals (CI) and Fisher's exact test using different models (dominant, recessive and additive) for dead and live mosquitoes in each treatment group are presented in Table 3. It was observed that F1534C-*kdr* conferred greater protection against DDT with all models and highest protection was shown using the recessive model (OR = 16.0, 95% CI: 5.6–45.4; *p* = 0.000). Lower protection was shown against deltamethrin when fitted with recessive (OR = 2.0, 95% CI: 1.06–3.75; *p*<0.05) or additive (OR = 1.85, 95% CI: 1.84–2.89; *p*<0.01) models. However, F1534C-*kdr* did not show significant protection against permethrin.

**Table 2:** Association of F1534 alleles with insecticide resistance phenotypes.

Insecticides		Genotype			Odds ratio (95% CI)			Fisher's exact test (*p* value)		
		FF	FC	CC	Recessive model	Dominant model	Additive model	Recessive model	Dominant model	Additive model
DDT	Dead	38	26	4	16 (5.64–45.42)	5.72 (3.18–10.30)	5.81 (3.76–8.96)	<0.0001	<0.0001	<0.0001
	Alive	41	72	113						
Deltamethrin	Dead	51	55	46	2.0 (1.06–3.75)	2.1 (0.99–4.33)	1.85 (1.84–2.89)	<0.05	NS	<0.01
	Alive	11	19	26						
Permethrin	Dead	50	59	99	1.05 (0.58–1.89)	0.77 (0.37–1.61)	1.37 (0.89–2.14)	NS	NS	NS
	Alive	9	18	31						

NS = non-significant.

**Table 3:** Genotyping results of PCR-RFLP assays for F1534C and T1520I alleles and their association.

		F1534 genotypes			
		FF	FC	CC	Total
T1520 genotypes	TT	28	22	105	155
	TI	0	8	37	45
	II	0	0	3	3
	Total	28	30	145	203

*p*HWE (Fisher's exact test): T1520 alleles = 0.991; F1534 alleles = 0.000.

### Genotyping of F1534 and T1520 alleles using new PCR-RFLP

Genotyping of F1534 and T1520 alleles were performed on 203 mosquitoes, which revealed a high frequency of the F1534C mutation (0.79) and a very low frequency of the T1520I mutation (0.13). Genotyping results showing association of T1520 and F1534 alleles are shown in Table 3. It was observed that T1520I mutation was found in individuals having the 1534C allele only, but never with wild type F1534. This data infers that 1520I is linked to 1534C. Linkage disequilibrium (LD) analysis revealed perfect disequilibrium (D′ = 1.0, χ^2^ = 8.02) though r^2^ was low (0.04) due to a relatively low frequency of allele 1520I as compared to 1534C, where all individuals with 1520I allele showed association with 1534C, but not all 1534C are associated with 1520I. The present data revealed the presence of three haplotypes with haplotype frequencies *f*_TF_ = 0.21, *f*_TC_ = 0.66 and *f*_IC_ = 0.13. However, *f*_IF_ was absent.

### Validation of new PCR-RFLP assays

Among the samples genotyped using the new PCR-RFLP, a portion of domain III was sequenced for 20 samples (two sample of TT/CC, five samples of TI/FC, eleven samples of TI/CC and two samples of II/CC). Genotyping results agreed with DNA sequencing results.

## Discussion

Vector control is the only option for suppression of *Ae. aegypti*-borne dengue and chikungunya infections in the absence of vaccine or drugs. Several pyrethroids have been recommended by WHO for use in space spray against *Aedes* [13]. Though in India pyrethroids are being extensively used in malaria control programme, their use in urban areas is limited to space spraying of pyrethrum and fogging with malathion. However, pyrethroid-based household anti-mosquito gadgets (liquid vaporizer, mats, coils) are extensively used as personal protectants against mosquito nuisance. Use of these devices may be contributing to resistance in *Ae. aegypti* in Delhi. Insecticide susceptibility tests carried out in India by several authors prior to the year 2014 did not reveal pyrethroid resistance, though they were found to be resistant to DDT [30]–[33]. Only in one case, 2% survival was recorded in *Aedes aegypti* on exposure to diagnostic concentration of deltamethrin in a strain from Jharkhand, India [30]. Very recently, for the first time in India, resistance to pyrethroids has been reported from Assam state [34].

In the absence of baseline insecticide susceptibility data for Indian *Ae. aegypti* or universally acceptable discriminating dose for *Ae. aegypti*, we used 4% DDT, 0.05% deltamethrin and 0.75% permethrin papers for bioassays, the most frequently cited doses in recent publications [30]–[31], [34]–[40] to facilitate easy comparison. Previous published data from Delhi showed that *Ae. aegypti* was 100% susceptible to even lower doses of insecticides, i.e., 0.025% deltamethrin and 0.25% permethrin [31]. Less than 80% mortalities of mosquitoes at higher doses (which are 2-fold and 3-fold respectively) confirm resistance against these insecticides.

The extensive use of insecticides for vector control has raised concern over the development of insecticide resistance and adverse effects on the environment and human health [41]. Genes conferring insecticide resistance have been spreading in vector populations, particularly in vectors of pathogens causing malaria and dengue [42]. The fact that dispersal of *Aedes* may be more rapid than other mosquitoes due to transportability of dried, but viable eggs through containers, a single resistance mechanism can spread rapidly. Knockdown resistance (*kdr*) is one of the mechanisms of DDT and pyrethroid resistance in insects. It is conferred by amino acid substitution(s) in the target site (VGSC) resulting in reduced sensitivity of the target site. A number of mutations have been reported in the VGSC of *Ae. aegypti* across Latin America and Southeast Asia amongst which V1016G/I, I1011M/V and F1534C [14], [18], [21], [22], [15], [43] are known to confer resistance. F1534C has been reported from Latin America [44], [22], [45] and Southeast Asia [15], [43], [46], and shown to confer resistance against DDT and pyrethroids. In our study we provide evidence that this mutation confers a high level of protection against DDT and relatively low protection against deltamethrin. However, we failed to show significant protection against permethrin. Our failure to establish association of F1534C with permethrin resistance is contrary to findings by Harris *et al.* (2010) [22] and Yanola *et al.*, (2011) [15]. Our result is also contradictory to the findings of Du *et al.*, (2013) [47] who were able to demonstrate that F1534C reduced the channel sensitivity to permethrin but not against deltamethrin when expressed in *Xenopus* oocyte. Failure to establish association of F1534C with permethrin resistance is surprising and needs to be further investigated. It may be possible that the dose of permethrin (0.75%) used for discrimination of *kdr*-resistant mosquito in the Indian population is too high that might have killed *kdr*-resistant mosquitoes. Another possible reason for such discrepancy may be due to the presence of some other linked mutations. For example, F1534C has shown to be strongly associated with permethrin in Grand Cayman [22] where another mutation V1016I co-existed. It is possible that protection against permethrin in Grand Cayman may be due to the combined effect of F1534C and V1016I. It is interesting to explore such association because such linkage has been shown in Brazil, where V1016I was always associated with F1534C [44]. To know the exact role of any particular *kdr* mutation, one should perform such association studies using laboratory lines of mosquitoes characterized for complete VGSC sequence.

In this study we explored a novel mutation T1520I in an Indian *Ae. aegypti* population. Its potential role in resistance to insecticides is yet to be ascertained. However, since this mutation has always been found in association with F1534C mutation (D′ = 1), it may be a compensatory mutation to reduce the fitness cost from possible deleterious effects of F1534C mutation, though it has been shown through laboratory experiments that *Ae. aegypti* homozygous for F1534C does not have reduced fitness [48]. However, its additive effect on protection against DDT and pyrethroids cannot be ruled out. We also observed that 1520I is associated with 1534C, but not vice versa explaining the low *r* ^2^ (0. 039), which reflects a very low frequency of T1520I as compared to F1534C. Whether haplotype 1534C/1520I is under positive selection remains to be established. Interestingly a similar linkage association is found in the Brazilian population where 1016I is associated with 1534C, but not vice versa [44]. These different associations in different geographical locations indicate that the most likely association of T1520I in India and V1016I in Brazil with F1534C are under positive selection. Linss *et al.* 2014 [44], noted a progressive increase of the Na_V_^R2^ haplotype (double mutant, F1534C with V1016I) from year 2002 through 2012 and concluded that it is likely to be the most favourably selected allele. Linkage of *kdr* mutations is very common in *Ae. aegypti*. Co-segregation has been shown between 1016G with D1794Y [17], 1016G with 989P [49] and 1016G with 1534C [22], [46]. Whether positive selection of such linkage associations is due to additive role in protection against insecticides or due to compensatory advantage, is worth investigating. Since novel mutation T1520I is tightly linked to F1534C and homozygotes are found in very low frequency, the exact role of this novel mutation could not be established.

Our result shows that F1534 genotypes show significant deviation from Hardy-Weinberg equilibrium in all populations (*p*<0.0001) except in South Delhi-II. Initially we thought that this might be due to discrepancy in allele specific PCR genotyping, which often fails to prevent non-specific annealing during PCR extension. However, when we carried out genotyping using highly specific PCR-RFLP method, there was no change in HWE parameter for F1534 alleles. Surprisingly, T1520 genotypes in the same group of mosquitoes were in perfect HWE (*p* = 0.99). The possible explanation for such a deviation may be the presence of heterogeneous populations or gene duplication. Further studies are required to resolve this issue.

Knockdown resistance, which is known to confer cross-resistance to DDT and pyrethroids cannot be monitored through routine insecticide susceptibility tests and requires a sensitive and reliable molecular method of detection. PCR-based methods, allele specific PCR in particular, are most widely used method for this purpose. The specificity of allele specific PCRs which are based on single nucleotide polymorphism is often compromised due to the fact that single base mismatch often does not prevent extension [50] and leads to non-specific amplification [26], [51]. Yanola *et al.*, (2011) reported an overestimation of 1534C frequency by 1.8% while using allele specific PCR [26]. In the present study, non-specific amplification was evident in the allele specific PCRs we employed for identification of various *kdr* alleles located at different loci. We therefore opted to develop a PCR-RFLP method for the identification of F1534 and T1520 alleles, which is presumed to be specific owing to the fact that restriction enzymes are highly specific. The sequencing of representative samples of PCR-RFLP genotyped samples (n = 20) showed 100% specificity of the assay. An additional advantage of the PCR-RFLP over allele specific PCR was that a single PCR amplicon could be used for detection of four alleles present at two loci since two mutations T1520I and F1534C are in proximity, whereas for allele specific PCR assays normally two PCR reactions are required to be performed for detecting four alleles at two loci.

The emergence of pyrethroid resistance in Indian *Ae. Aegypti* associated with the presence of the F1534C-*kdr* mutation is a threat to the success of pyrethroid-based *Aedes* control and necessitates countrywide monitoring of insecticide resistance and mapping the distribution of F1534C and T1520I mutations. Though F1534C mutation is shown to be associated with DDT and pyrethroids, the role of novel mutation T1520I still remains to be investigated.

## Supporting Information

S1 Fig Geographical locations of mosquito collection sites.
(PDF)

S1 Table Geographical locations and dates of mosquito collection.
(DOCX)
