# Supplementary figures and images for: Pyrethroid-Resistance and Presence of Two Knockdown Resistance (kdr) Mutations, F1534C and a Novel Mutation T1520I, in Indian Aedes aegypti
Source: PLoS Negl Trop Dis. 2015 Jan 8;9(1):e3332. doi: 10.1371/journal.pntd.0003332 (PMC4287524; doi:10.1371/journal.pntd.0003332)

Figure S1: Geographical locations of mosquito collection

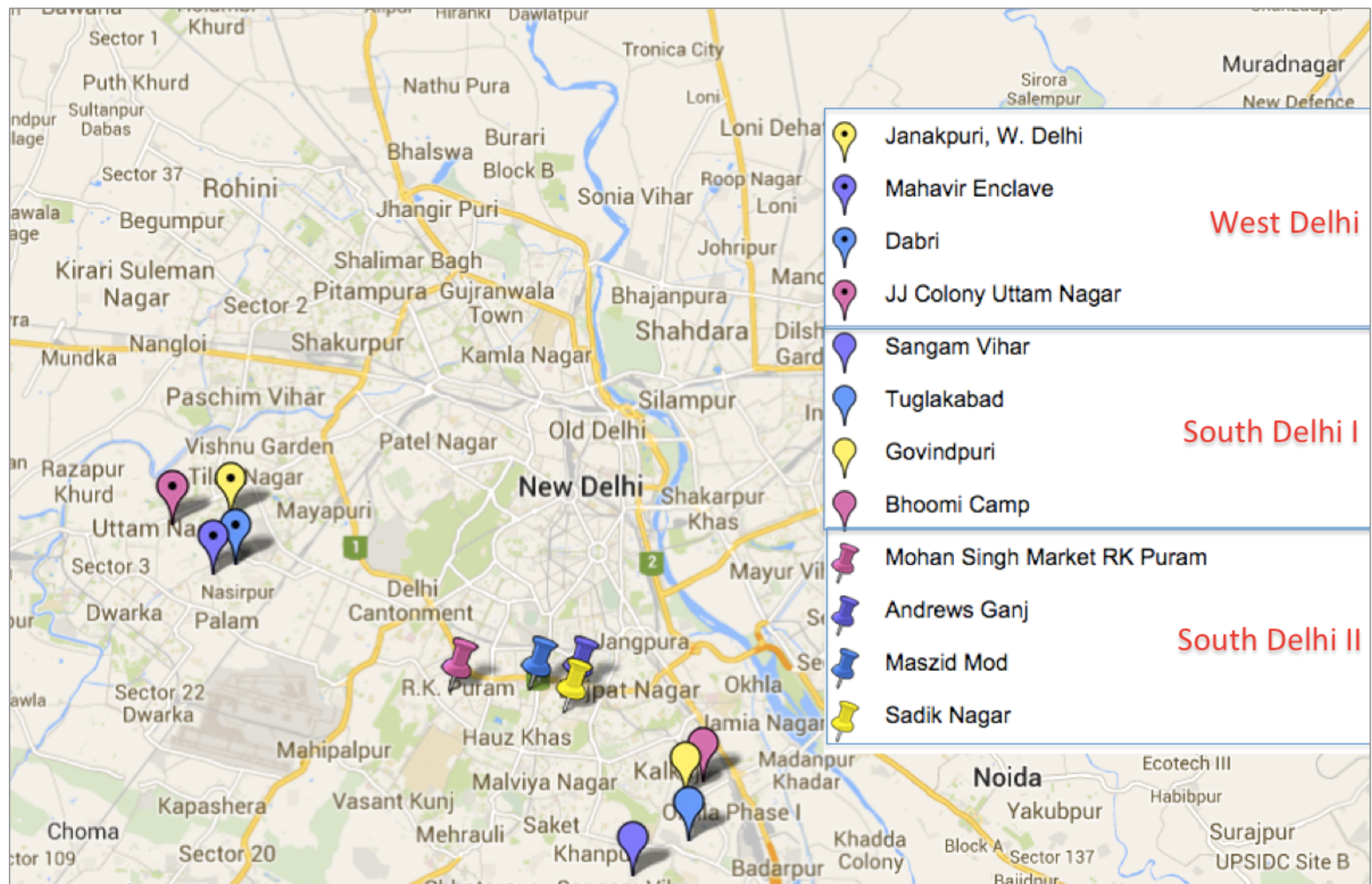

Supplement: S1 Fig — Geographical locations of mosquito collection sites. (PDF) [file pntd.0003332.s001.pdf]
