# Supplementary material for: Pyrethroid-Resistance and Presence of Two Knockdown Resistance (kdr) Mutations, F1534C and a Novel Mutation T1520I, in Indian Aedes aegypti
Source: PLoS Negl Trop Dis. 2015 Jan 8;9(1):e3332. doi: 10.1371/journal.pntd.0003332 (PMC4287524; doi:10.1371/journal.pntd.0003332)
Supplement: S1 Table — Geographical locations and dates of mosquito collection. (DOCX) [file pntd.0003332.s002.docx]

Table S1: Geographical locations and dates of mosquito collection

| Locality | GPS Coordinates | | Collection dates (Year 2012) | | | | | |
| --- | --- | --- | --- | --- | --- | --- | --- | --- |
|  | Longitude | Latitude | MAY | JUN | JUL | AUG | SEP | OCT |
| ***West Delhi*** | | | | | | | | |
| Janakpuri | 28.62 | 77.08 | 1st | 7th | ND | 12th | 11th | ND |
| JJ Colony Uttam Nagar | 28.62 | 77.07 |  |  |  |  |  |  |
| Mahavir Enclave | 28.59 | 77.08 |  |  |  |  |  |  |
| Dabri | 28.61 | 77.08 |  |  |  |  |  |  |
| ***South Delhi I*** | | | | | | | | |
| Sangam Vihar | 28.50 | 77.25 | 10th | 4th | 3rd  &  12th | 3rd | 12th | 9th |
| Tughalkabad | 28.51 | 77.26 |  |  |  |  |  |  |
| Govindpuri | 28.53 | 77.26 |  |  |  |  |  |  |
| Bhoomi Camp | 28.53 | 77.27 |  |  |  |  |  |  |
| ***South Delhi II*** | | | | | | | | |
| Sadik Nagar | 28.56 | 77.22 | 11th | 5th | 4th | 1st | 6th | 10th |
| Andrewj Ganj | 28.56 | 77.23 |  |  |  |  |  |  |
| Maszid Mod | 28.56 | 77.22 |  |  |  |  |  |  |
| Mohan Singh Mkt, RK Puram | 28.57 | 77.17 |  |  |  |  |  |  |

ND= not done
